# Affinity Constants of Bovine Serum Albumin for 5 nm Gold Nanoparticles (AuNPs) with ω-Functionalized Thiol Monolayers Determined by Fluorescence Spectroscopy

**DOI:** 10.1021/acs.langmuir.4c01234

**Published:** 2024-07-16

**Authors:** Jennifer L. Hanigan-Diebel, Robert J. Costin, Logan C. Myers, Christopher I. Vandermeer, Miles S. Willis, Kiran Takhar, Ogechukwu V. Odinakachukwu, Matthias G. Carroll, Jarrod E. Schiffbauer, Samuel E. Lohse

**Affiliations:** δ Chemistry Department, Central Washington University, 400 East University Way, Ellensburg, Washington 98926, United States; † Department of Physical and Environmental Sciences, Colorado Mesa University, 1100 North Ave, Grand Junction, Colorado 81501, United States

## Abstract

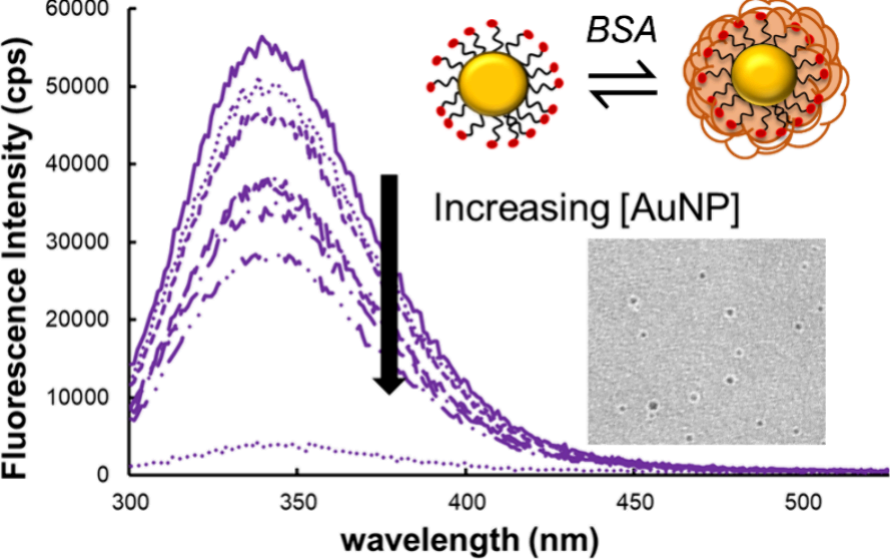

A detailed understanding of the binding of serum proteins to small (*d*_core_ <10 nm) nanoparticles (NPs) is essential for the mediation of protein corona formation in next generation nanotherapeutics. While a number of studies have investigated the details of protein adsorption on large functionalized NPs, small NPs (with a particle surface area comparable in size to the protein) have not received extensive study. This study determined the affinity constant (*K*_a_) of BSA when binding to three different functionalized 5 nm gold nanoparticles (AuNPs). AuNPs were synthesized using three ω-functionalized thiols (mercaptoethoxy–ethoxy–ethanol (MEEE), mercaptohexanoic acid (MHA), and mercaptopentyltrimethylammonium chloride (MPTMA)), giving rise to particles with three different surface charges. The binding affinity of bovine serum albumin (BSA) to the different AuNP surfaces was investigated using UV–visible absorbance spectroscopy, dynamic light scattering (DLS), and fluorescence quenching titrations. Fluorescence titrations indicated that the affinity of BSA was actually highest for small AuNPs with a negative surface charge (MHA-AuNPs). Interestingly, the positively charged MPTMA-AuNPs showed the lowest *K*_a_ for BSA, indicating that electrostatic interactions are likely not the primary driving force in binding of BSA to these small AuNPs. *K*_a_ values at 25 °C for MHA, MEEE, and MPTMA-AuNPs were 5.2 ± 0.2 × 10^7^, 3.7 ± 0.2 × 10^7^, and 3.3 ± 0.16 × 10^7^ M^–1^ in water, respectively. Fluorescence quenching titrations performed in 100 mM NaCl resulted in lower *K*_a_ values for the charged AuNPs, while the *K*_a_ value for the MEEE-AuNPs remained unchanged. Measurement of the hydrodynamic diameter (*D*_h_) by dynamic light scattering (DLS) suggests that adsorption of 1–2 BSA molecules is sufficient to saturate the AuNP surface. DLS and negative-stain TEM images indicate that, despite the lower observed *K*_a_ values, the binding of MPTMA-AuNPs to BSA likely induces significant protein misfolding and may lead to extensive BSA aggregation at specific BSA:AuNP molar ratios.

## Introduction

Noble metal nanoparticles are intriguing prospective biomedical nanotheranostic agents, due to their unique size- and shape-dependent optical properties.^1−8^ The specific size-dependent optical properties that metal nanoparticles manifest are governed by the composition, size, and shape of the core material, and these properties can be leveraged for many pertinent applications in ways that molecular pharmaceuticals and imaging agents cannot.^1−8^ Particularly, gold nanoparticles' (AuNPs') plasmonic properties can be used to enable high-resolution imaging applications (e.g., two-photon luminescent imaging with high resolution),^1−3^ targeted drug delivery,^1−3^ and noninvasive therapies (such as photothermal therapy).^6,8−10^ Furthermore, as a result of their particular size regime (at least one dimension of 100 nm or less), NPs are capable of interacting in unique ways with tissues, cells, and even specific biological molecules.^1,2,5,8−10^ Despite the potential of metal NP’s size-dependent properties, and despite a surge of research into the *in vitro* and *in vivo* interactions of metal NPs with biological systems with various stages of complexity, there are still key gaps in our understanding of how NPs interact with tissues, cells, and even individual proteins.^1,2,9−12^

The successful application of nanotherapeutics (at minimum) relies on very precise control of the nanomaterial’s physiochemical properties (size, shape and surface chemistry), both to ensure that the NP possesses the necessary size-dependent properties, and to make sure that the NP targets and distributes in the patient correctly.^1,2,9,10^ In addition to the synthetic challenge of preparing nanotheranostic agents with precise physiochemical properties, *in vivo* applications of metal nanoparticles are further complicated by the fact that the “synthetic identity” of the NP is distinct from the “biological identity” that the NP takes on as soon as it enters an organism.^13−17^ The biological identity of the particle encompasses all the physiological changes that occur (size, aggregation state, adsorption of biomolecules, etc.) when the NP is exposed to a biological environment.^13−15,18−31^ The best known physiochemical transformation associated with the biological identity of a NP is the formation of the protein corona (PC), a strongly adsorbed shell of serum proteins that bind to the NP as soon as it enters the bloodstream.^10,13,15,19−31^ The composition of the protein corona is influenced by both the original physiochemical properties of the NP and the biological compartments that the NP enters. The job of a nanotherapeutic is therefore made more difficult by the fact that nanomaterials are changed by the biological fluids that they encounter.

The PC becomes the new biological identity of the NP, dictating (for better or worse) its interactions with its surroundings.^10,13,15,23^ The formation of the NP’s PC is driven by a variety of intermolecular interactions, including van der Waals forces, hydrogen bonding, electrostatic forces, and hydrophobic interactions.^10,15^ The dominant intermolecular forces depend on the NP’s physiochemical characteristics (primarily size and surface chemistry), although electrostatic interactions are generally believed to be a major driving force.^10,15,23,30,31^ Because of its pivotal role in determining the biological identities of engineered NPs, the formation of the PC has been extensively researched, both with respect to the binding strength of individual proteins (particularly albumin)^18,21,24−28^ and with respect to the composition of the PC (particularly PCs resulting from whole serum).^15,20,23,29−31^ The vast majority of this research has focused on NPs with diameters (50–200 nm) significantly larger than those of the proteins with which they are interacting with. Traditionally, the protein corona is understood to form in two empirically defined layers: the tightly associated “hard” corona at the NP surface and the loosely associated “soft” corona, which forms above the hard corona.^10,13,15,23^ Recent research has revealed that the structure of the protein corona depends on the relative size of the AuNP compared to the size of the protein binding to the NP surface.^20,28,30^ Piella et al. studied the formation of albumin corona formation of citrate-capped AuNPs ranging in size from 3.5 to 150 nm.^20^ For the smallest AuNPs, which are smaller than the serum proteins with which they interact, the PC remained loosely and incompletely formed. As the NP core size increased, PC formation became dense and less labile. Indeed, for the largest particles, a multilayer PC was observed.^20^

While these studies have revealed much about the size-dependent aspects of protein corona formation, the role of the NP’s surface chemistry in controlling binding interactions with serum proteins has received significantly less investigation, particularly for smaller NPs (*d*_core_ <10 nm). Small NPs typically show better tissue and cell permeability, as well as more complete renal clearance, with a longer lifetime in the circulatory system.^1,2,9,10,13,15^ Therefore, NPs with core diameters less than 10 nm are attracting more attention as potential nanotherapeutics, and elucidating their interactions with serum proteins has become more pressing. Specifically, understanding the affinity of individual proteins for different NP surfaces helps clarify how these small NPs are viewed by specific proteins in serum and is an essential steppingstone to the systematic understanding of the bio transformations of these small NPs. The affinity of specific proteins for NPs can be quantified using a variety of instrumental techniques.^21,22,24−28^ Spectroscopic techniques (UV–vis absorbance spectroscopy, dynamic light scattering (DLS), fluorescence quenching titration, etc.) can be used to determine affinity constants (*K*_a_) for specific proteins interacting with NPs that possess appropriate optical properties (most commonly gold nanoparticles, AuNPs).^1,20−22,24−26^ Affinity capillary electrophoresis (ACE) and isothermal titration calorimetry (ITC) have also been used as a more general method to determine the affinity of proteins for NP surfaces, regardless of the optical activity of the NPs.^21,27^

Among the NP probes available to study the thermodynamics and kinetics of protein-NP binding interactions, AuNPs are one of the most versatile.^1^ In addition to their potential significance as theranostic agents, their strong plasmonic properties make AuNPs ideal for investigating the influence of nanoparticle physiochemical properties on their biological interactions using spectroscopic techniques.^1,4,5,21,26^ Furthermore, AuNPs are generally more stable than other NP core materials and a well-established library of synthetic techniques exist to tailor their original physiochemical properties (including size, shape, and surface chemistry).^1,4,5,25,26,28^ The direct synthesis of ω-thiol-stabilized gold clusters, in particular, is an established technique that allows for the synthesis of small gold nanoparticles (*d*_core_ ≤5 nm), with precisely tailored surface chemistries.^32−36^ This synthetic approach provides a facile route to AuNPs with identical sizes but different surface charges and potentially extends to the synthesis of nanomaterial probes with mixed ligand shells.^1,4,5,33,36^ The strong absorbance and light scattering properties of these AuNPs mean that *K*_a_ values can be determined using a variety of simple spectroscopic techniques.^19,25,26,28^

**Figure 1:**
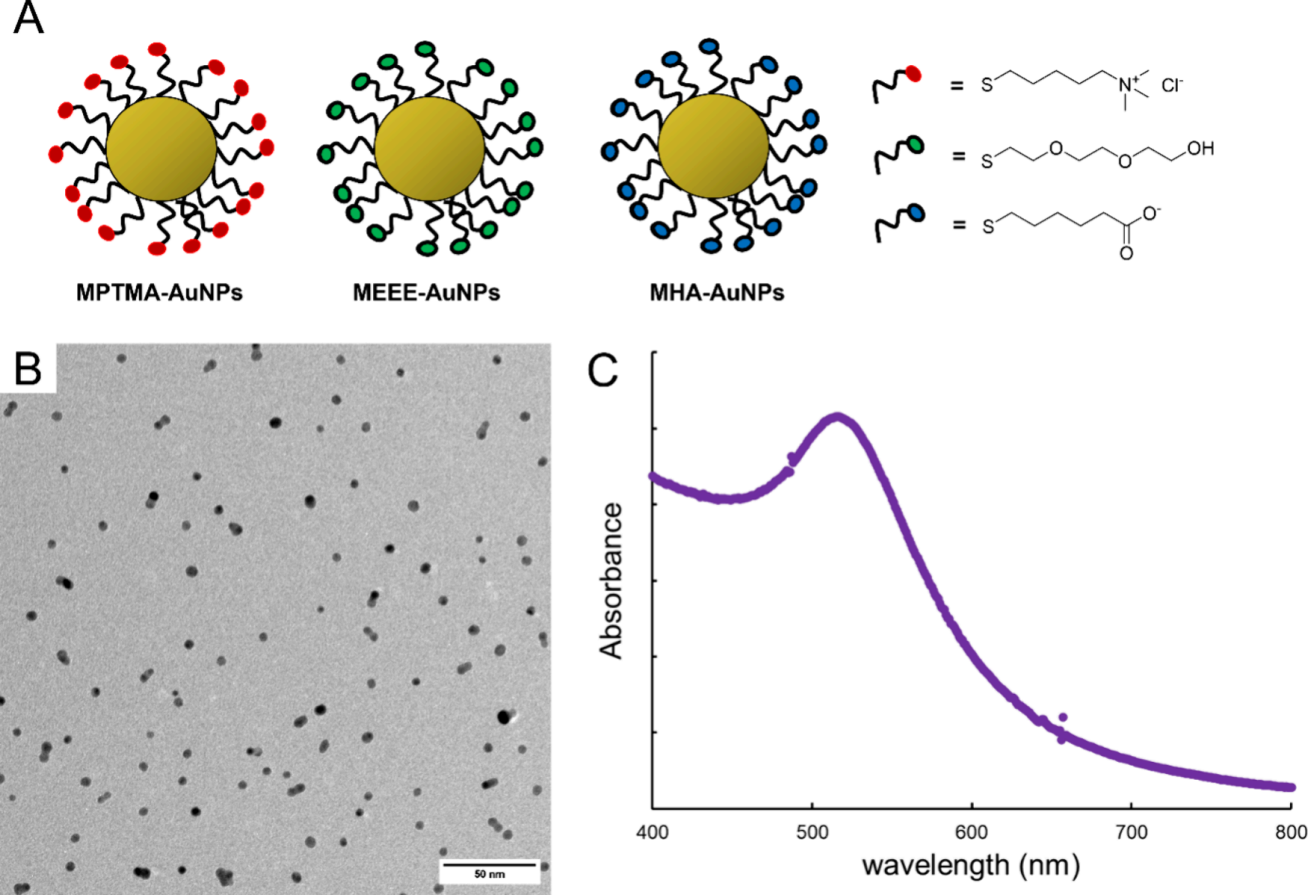
Functionalized AuNP probes used in this study. (A) Schematic representations of the functionalized 5 nm AuNPs. (B) Representative TEM micrograph of the 5 nm AuNPs. Scale bar is 50 nm. (C) Representative absorbance spectrum of aqueous 5 nm AuNP dispersion.

In this study, the binding interactions between different 5 nm thiol-stabilized AuNP surfaces and bovine serum albumin were investigated by using UV–vis absorbance spectroscopy, dynamic light scattering, and fluorescence quenching titrations. A library of 5 nm AuNPs displaying a negatively charged surface (mercaptohexanoic acid (MHA)), a neutral surface (mercapto-ethoxy-ethoxy-ethanol (MEEE)), and a positively charged surface (mercaptopentyltrimethylammonium bromide (MPTMA)) were prepared using a direct synthesis method.^35,36^ This library of 5 nm AuNPs was used to determine how surface charge mediates the binding interaction between the small AuNPs and BSA (Figure 1). Albumin is the most abundant serum protein (MW = 67 kDa, pI = 4.7, hydrodynamic diameter (*D*_h_ ∼7 nm)) and is similar in overall size to the AuNPs being investigated.^19^ BSA is often used as an albumin model in protein-NP binding studies, because BSA has many structural similarities and high sequence homology to human serum albumin (HSA).^18,19,21,22^ These small NP probes may open up unique interactions between the protein and nanoparticle surfaces, which have seldom been investigated in other studies of protein binding.^18,25,26,28,37,38^ The variation in the surface chemistry of the AuNP probes is illustrated in Figure 1A. The physiochemical properties of BSA-AuNPs conjugates were studied quantitatively using UV–vis absorbance spectroscopy, dynamic light scattering (DLS), and transmission electron microscopy (TEM). Ultimately, affinity constants (*K*_a_) were obtained by fluorescence quenching titrations in both water and a sodium chloride solution (100 mM NaCl) at 25 °C.

## Experimental Section

### Materials

Hydrogen tetrachloroaurate trihydrate (HAuCl_4_·3H_2_O, 97%) was obtained from Aldrich, sodium borohydride (NaBH_4_), sodium hydroxide (NaOH), sodium borohydride (NaBH_4_), sodium thiosulfate (Na_2_S_2_O_3_), 5-bromo-pentytrimethylammonium bromide (97%), 6-bromohexanoic acid (97%), 2-[2-(2-chloroethoxy)ethoxy] ethanol (97%), iodine (I_2_, 97%), deuterium oxide (D_2_O), bovine serum albumin (>98%), sodium bicarbonate (Bioreagent), hydrochloric acid (37% w:w, ACS Reagent), and sodium chloride (>99%) were obtained from Sigma and used as received. Sodium bicarbonate buffer was prepared from sodium bicarbonate solid, and buffer pH was adjusted by addition of hydrochloric acid (HCl(*aq*)). Milli-Q deionized water (18 MΩ) was used as a solvent in all stock solution preparations.

pH measurements were performed with a Mettler-Toledo, SG23 hand-held pH meter. Absorbance spectra were recorded using an Agilent 8543 UV–visible spectrophotometer. Dynamic light scattering (DLS) analysis was performed using a Malvern ZetaSizer 900. ^1^H NMR spectroscopy was performed on a Bruker 400 MHz NMR spectrometer. Fluorescence spectroscopy was performed on a Horiba FluoroMax fluorometer. Fluorescence cuvettes were obtained from Alpha Nanotech. Transmission electron microscopy (TEM) images of the purified particles were obtained using a Thermo FEI Tecnai G2 Spirit Transmission Electron Microscope operated at 300 kV. Negative-stain TEM images were obtained using a Titan Krios G3i Transmission Electron Microscope, and protein-AuNP conjugates were visualized using a uranyl acetate stain. Pall Tangential flow filtration cassettes (diafiltration of 10 kDa) were obtained from VWR.

### Functionalized Bunte Salt Preparation

Functionalized Bunte salt ligand precursors (the mercapto ethoxy–ethoxy–ethanol Bunte salt analog (MEEE BS), the mercapto hexanoic acid Bunte salt analog (MHA BS), and the mercapto pentyltrimethylammonium Bunte salt analog (MPTMA BS)) were synthesized according to previously published methods.^35,36^ Briefly, organohalide precursors 2-[2-(2-chloroethoxy)ethoxy] ethanol (for MEEE BS), 5-bromopentyltrimethylammonium bromide (for MPTMA BS), or 6-bromohexanoic acid (for MHA BS) and sodium thiosulfate (Na_2_S_2_O_3_) were combined in a 1:0.8 molar ratio in a 50:50 solution of Milli-Q H_2_O and absolute ethanol and refluxed for at least 4 h.^35^ The water and ethanol were removed by rotary evaporation. The crude product was redissolved in ethanol and gravity filtered. Then, the ethanol was removed once again by rotary evaporation followed by overnight drying in a vacuum oven.^35,36^ Formation of the functionalized Bunte salts was verified by ^1^H NMR spectroscopy (D_2_O, 400 MHz).

### AuNP Synthesis and Purification

AuNPs were synthesized according to previously published methods.^35,36^ Briefly, 5 mol equiv HAuCl_4_ was mixed with 2 mol equiv functionalized Bunte salt (either MEEE BS, MHA BS, or MPTMA BS) in water and stirred with a magnetic stir bar and plate for 5 min.^35^ Ice-cold aqueous sodium borohydride (2 mol equiv) was then added to the HAuCl_4_ solution, and stirring was continued. Desired AuNP core sizes were achieved by adjusting pH of both solutions with small amounts of 1 M NaOH and by adjusting the concentrations of Bunte salts used. Functionalized AuNPs were then purified via diafiltration.^39^ Prior to diafiltration, the crude AuNP dispersion was reduced to an initial volume of 15 mL in the diafiltration apparatus. Then, Milli-Q water was added to the AuNP dispersion as diafiltration continued until 1 L of total filtrate volume (66 vol equiv) had been passed through the system.

### Purified AuNP Characterization

AuNP core sizes (*d*_core_) and AuNP dispersion’s molar concentrations were determined via UV–visible absorbance spectroscopy according to previously published methods.^40^ The AuNP core size was determined using the ratio of the NP solution’s absorbance at the SPR band (*A*_spr_) versus the absorbance at 450 nm (*A*_450_), using the model developed by Haiss et al.^40^
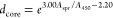
1

For TEM imaging, aqueous solutions of the AuNPs were drop-cast directly onto Cu/SiO TEM grids (PELCO) for imaging. ImageJ software (FIJI) was used to analyze the TEM images to determine the average core diameter. The hydrodynamic diameter (*D*_h_) of the purified AuNPs in solution was determined by dynamic light scattering (DLS). The surface composition of the ligands on the purified nanoparticle was determined by ^1^H NMR spectroscopy (D_2_O, 400 MHz), after digesting the AuNPs with diiodide by addition of solid diiodide to the NMR tubes containing the AuNP sample.^41^

### BSA-AuNP Binding Investigations Using UV–Vis Absorbance Spectroscopy

Absorbance spectroscopy data for purified functionalized AuNPs were obtained in aqueous AuNP dispersions at [AuNP] = 50.0 nM in bicarbonate-buffered solutions (pH = 7.4) incubated at 25 °C for 90 min. Absorbance spectra were then obtained while increasing the [BSA] from 0.0 to 7.0 mg/mL BSA across multiple samples at a constant [AuNP] of 50.0 nM. Absorbance data were obtained in triplicate.

### BSA-AuNP Binding Investigations by DLS Analysis

DLS data for purified functionalized AuNPs were obtained in aqueous AuNP dispersions at a [AuNP] = 500.0 nM in bicarbonate-buffered solutions (pH = 7.4) incubated at 25 °C for 90 min. DLS determinations of the hydrodynamic diameter (*D*_h_) for BSA-AuNP complexes were performed by increasing the [BSA] from 0.0 to 7.0 mg/mL BSA across multiple samples at a constant [AuNP] of 500.0 nM. DLS data were obtained in triplicate for each sample, and in spectra where multiple scattering peaks were observed, the lowest average diameter peak (*D*_h_ ∼10 nm) was used to obtain the primary particle size. DLS analysis was performed at 25 °C in Milli-Q water at pH = 7.4.

### BSA-AuNP Binding Constant Measurements by Fluorescence Titration

Bovine serum albumin (0.44 mg/mL) was incubated with AuNPs in varying concentrations from 0 to 30 nM in bicarbonate-buffered solutions at pH 7.4 for 90 min at 25 °C. Sample tubes and BSA solutions were kept covered in foil and in the dark at all times prior to analysis. After 90 min of incubation, fluorescence spectroscopy was performed with excitation at 280 nm and emission detected from 300 to 525 nm. The quartz cuvette was rinsed once with ethanol and twice with Milli-Q water between each sample. The fluorescence emission intensity was measured at 350 nm to determine the fluorescence intensity in both the presence and absence of AuNPs. Fluorescence titrations were performed in both Milli-Q water and 100 mM aqueous sodium chloride. All fluorescence titration measurements were performed in triplicate.

Stern–Volmer plots were created by plotting the ratio of the emission in the absence of AuNPs (*F*_0_) over emission at the [AuNP] of interest (*F*) versus AuNP concentration. The loss of BSA’s intrinsic fluorescence due to quenching is related to quencher concentration [*Q*] (in this case, [AuNP]) by the Stern–Volmer equation:^21,26,28^
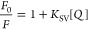
2

Assuming static quenching, the Stern–Volmer constant is considered the affinity constant, *K*_a_.^21,28^

Cooperativity in binding of BSA to the AuNP surface was determined by constructing a Hill Plot. Hill plots were created by plotting the log of ((*F*_0_*– F*)/*F – F*_sat_)), where *F*_sat_ is fluorescence at saturation, versus the log of AuNP concentration. The slope of the Hill Plot (*n*) is a measure of the cooperativity in BSA-AuNP binding.^21^ The Hill Plot takes the form of the equation below:
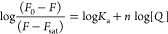
3

## Results and Discussion

### AuNP Characterization

Prior to investigating the binding behavior between small functionalized AuNPs, the size of the AuNPs was characterized using a combination of absorbance spectroscopy, TEM, and DLS. When the NP core consists of gold, absorbance spectroscopy can be used to characterize core diameter (*d*_core_).^40^ The ratio of absorbance at the plasmon peak (*A*_spr_) and absorbance at 450 nm (*A*_450_) gives the average core diameter.^40^ An example absorbance spectrum is shown in Figure 1C. AuNP core size was confirmed by TEM imaging (Figure 1B, Figures S1 and S2), and DLS was used to determine the hydrodynamic diameter (*D*_h_) of the functionalized AuNPs. The size characterization data obtained from all the techniques are summarized in Table 1. The size characterization data indicate that the AuNPs synthesized are indeed slightly less than 5 nm in core diameter, with an overall hydrodynamic diameter (including the functionalized thiols) of ∼10 nm, regardless of the ligands displayed by the AuNP.

**Table 1:** Size Characterization Data for the AuNP Probes

**AuNP surface chemistry**	**UV–vis core size (nm)^a^**	**TEM *d***_**core**_**(nm)**	*D*_h_**(nm)**
MPTMA	3.9	4.8 ± 1.7 (*n* = 1679)	9.4 ± 1.6
MEEE	4.3	4.7 ± 2.2 (*n* = 779)	9.8 ± 1.7
MHA	5.0	4.9 ± 2.0 (*n* = 519)	10.3 ± 1.6

a In this table, UV–vis core size represents the core diameter determined from the absorbance spectrum of the aqueous AuNP dispersion,^40^ while *d*_core_ represents the core size determined from size analysis of the TEM images.

### Determination of Surface Ligand Composition by ^1^H NMR Spectroscopy

To ensure that the AuNPs were synthesized with the desired surface chemistries, ^1^H NMR spectroscopy (400 MHz) was performed on iodine-digested solutions of the purified AuNPs redispersed in D_2_O.^41^ The ^1^H NMR spectra indicate that the MPTMA-, MEEE-, and MHA-AuNPs display the expected thiol ω-functionalities on their surfaces. Figures S3–S6 show the ^1^H NMR spectra of the MEEE-capped AuNPs, MHA-capped AuNPs, and MPTMA-capped AuNPs (Supporting Information, Figures S3–S6).

### Characterization of BSA-AuNP Conjugate Formation

**Figure 2:**
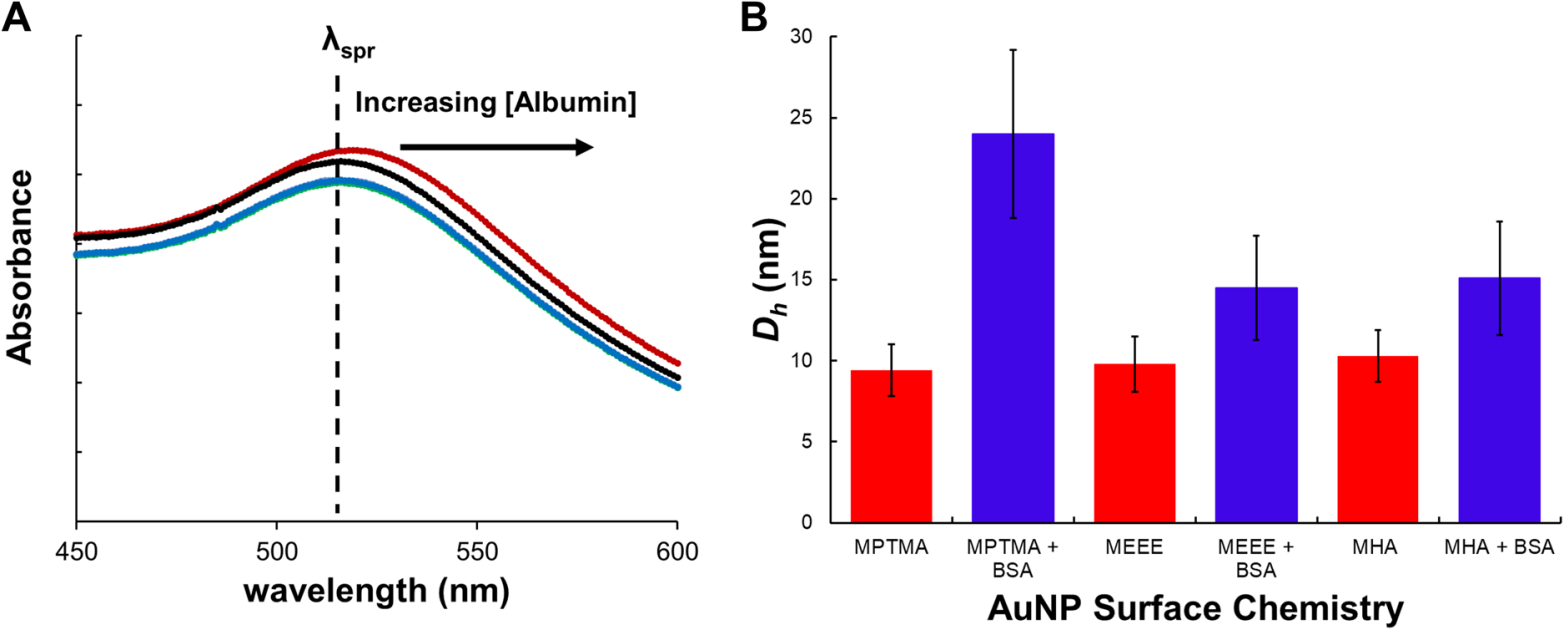
(A) Representative absorbance spectra showing wavelength shifts for solutions of MEEE-AuNPs in the presence of increasing [BSA], pH = 7.4, and [AuNP] = 50 nM. (B) Summary of DLS data for the functionalized AuNPs in the absence of BSA (red) and in the presence of BSA (blue) at a pH = 7.4, [BSA]:[AuNP] = 1.5:1, [NaCl] = 0 mM, and [AuNP] = 500 nM.

Prior to studying the binding interactions of the functionalized AuNP probes with BSA, we sought to establish the incubation time required for the 5 nm AuNPs to form stable conjugates with BSA and to characterize the physiochemical properties of the BSA-AuNP conjugates. The BSA-AuNP physiochemical properties were characterized using dynamic light scattering, absorbance spectroscopy, and negative-stain TEM imaging. The binding of BSA to the AuNP surface was investigated in solutions buffered with bicarbonate buffer (pH = 7.4) at [AuNP] = 50.0 nM (absorbance spectroscopy studies) and [AuNP] = 500.0 nM (DLS studies). A higher [AuNP] had to be employed in the DLS studies to make sure that sufficient light scattering signal was obtained from the smaller particles. The formation of the BSA-AuNP conjugates was characterized both in Milli-Q water and in 100 mM aqueous NaCl. These studies indicated (as expected) that BSA successfully forms conjugates with all of the AuNP surface chemistries investigated here. An incubation time of 60 min was found to be sufficient for the BSA-AuNP complexes to reach equilibrium, as determined by tracking the change in the λ_spr_ for the AuNP dispersions following BSA exposure. The time-resolved UV–visible absorbance spectra of the functionalized AuNPs taken in both water and aqueous sodium chloride solution (100 mM NaCl) indicate that BSA binds to the functionalized AuNPs quickly, with an equilibrium binding condition being established rapidly (within 90 min; see Figure S7). The successful formation of the AuNP-BSA conjugate is indicated by a small red-shift (Δλ ∼2 nm) compared to the λ_spr_ of the AuNP dispersion prior to protein exposure (Figure 2A). These rapid BSA-AuNP conjugate formation kinetics are consistent with a majority of previous studies, which have determined that BSA (or other proteins) can saturate the NP surface and form a stable complex in approximately 20 min of exposure time.^21,26,28,42^

Having established the time frame required to form the BSA-AuNP conjugates, we next attempted to use absorbance spectroscopy and dynamic light scattering titration to characterize a typical BSA-AuNP conjugate. In these experiments, the [AuNP] was held constant, while the [BSA] concentration was systematically increased.^20,21,26,28^ The Δλ_max_ for the AuNP dispersion’s (absorbance titration) or Δ*D*_h_ was then monitored as a function of [BSA]:[AuNP]. [BSA]:[AuNP] conditions that show saturation of the Δλ_max_ or Δ*D*_h_ were used to qualitatively characterize the size of the BSA-AuNP conjugates, and hence, the number of BSA molecules likely bound to the AuNP. The key assumption here is that adsorption of the albumin to the AuNP surface changes the dielectric environment around the particle surface, and thus, the λ_spr_ changes until the surface of the AuNP has been saturated by BSA. Once the surface of the AuNP has been saturated by BSA, the change in λ_spr_ also begins to saturate.^20,21,26,28^ Both the absorbance spectroscopy and DLS data confirm that the binding of one or two BSA molecules is sufficient to saturate the surface of the MEEE- and MHA-AuNPs. The characteristic Δλ_max_ of AuNP-BSA complex formation saturates even when there are equimolar amounts of BSA and the AuNPs (Figure 2A and Figure S8). DLS data further support this, with the *D*_h_ changing by ∼5 nm (the approximate *D*_h_ of one BSA protein in its native conformation) upon exposure of the MEEE- or MHA-AuNPs to roughly equimolar amounts of BSA (Figure 2B).^20,21,26,28^ A key exception here would be the MPTMA-AuNPs, whose *D*_h_ changes by nearly 15 nm upon exposure to equimolar BSA, which would be consistent (based on the hydrodynamic diameter change) with 2–3 proteins associating with the MPTMA-AuNPs under these conditions. Extensive BSA misfolding leading to heteroaggregation of the MPTMA-AuNPs with BSA would be another route to this substantially larger *D*_h_. Our observations support a picture (similar to previous studies of 3–5 nm citrate-AuNPs exposed to BSA) that the small AuNPs form a conjugate (“loose corona”) with several BSA molecules, as opposed to a hard protein corona.^20^ It should also be emphasized here that a true protein corona involves numerous serum protein components and biomolecules, as opposed to our system, where only a single “model” protein has adsorbed to the AuNP surface. Hence, when we refer to the corona acquired by the 5 nm AuNPs in this study, we are only referring to the adsorbed BSA. Intrigued by the larger Δ*D*_h_ observed for the MPTMA-AuNPs at roughly equimolar [BSA]:[AuNP] ratios, we attempted to more systematically characterize the BSA-AuNP conjugates that form at this BSA:AuNP ratio, using a combination of absorbance spectroscopy, DLS, and TEM imaging.

**Figure 3:**
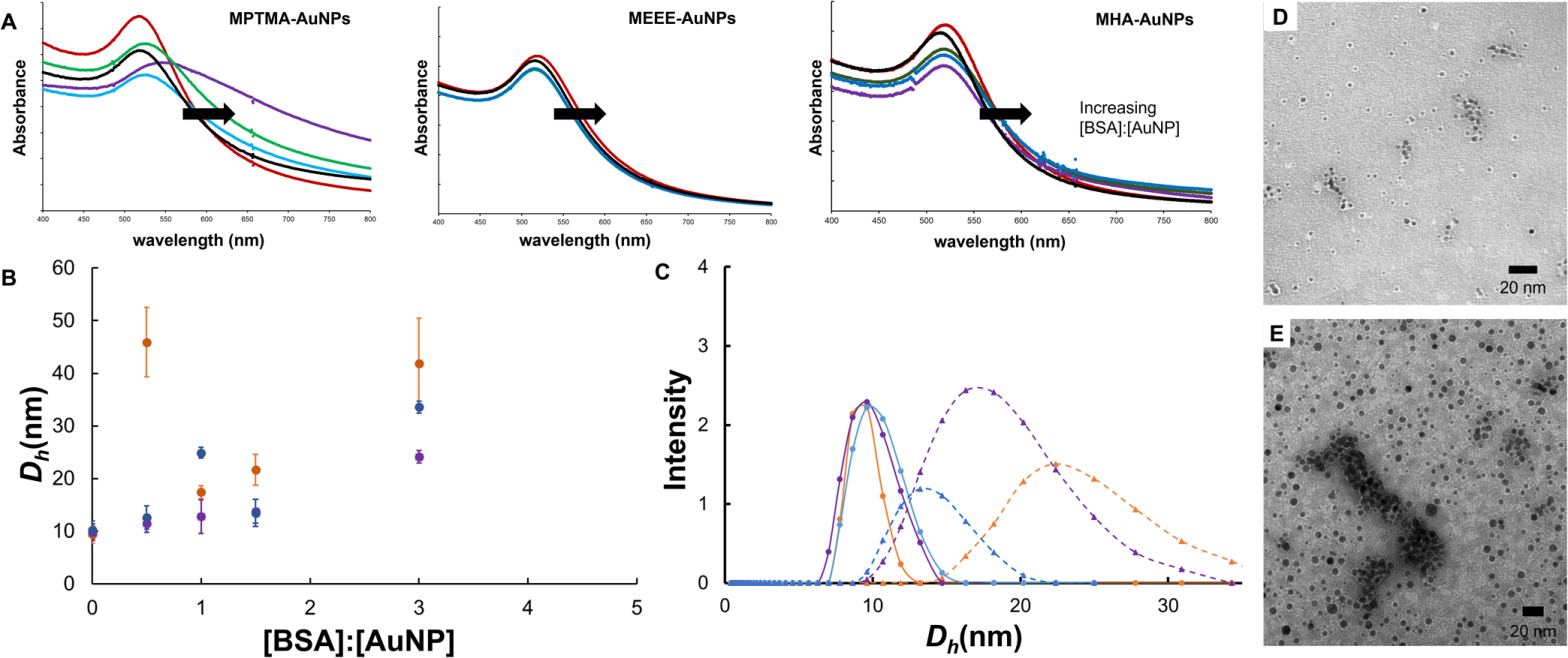
(A) Representative absorbance spectra showing wavelength shifts for solutions of MPTMA-, MEEE-, and MHA-AuNPs in the presence of increasing [BSA], [AuNP] = 50 nM. Black trace (0 BSA:AuNP molar ratio), blue trace (0.5:1 BSA:AuNP), green trace (0.8:1 BSA:AuNP), purple trace (4:1 BSA:AuNP), and red trace (50:1 BSA:AuNP). (B) Summary of DLS data for the functionalized AuNPs in the presence of increasing [BSA] at pH = 7.4, [NaCl] = 0 mM, and [AuNP] = 500 nM. Orange = MPTMA-AuNPs, purple = MEEE-AuNPs, and blue = MHA-AuNPs. (C) Normalized DLS Intensity plots for AuNP dispersions before and after exposure to 50:1 [BSA]:[AuNP]. Spheres represent AuNP dispersions before protein exposure, and triangles represent AuNP dispersion after protein exposure. (D, E) Uranyl-acetate-stained TEM images of MEEE-AuNPs and MPTMA-AuNPs incubated with BSA at [BSA]:[AuNP] 1.5:1. Scale bars are 20 nm.

To visualize the BSA-AuNP conjugates, the complexes (prepared at a molar BSA:AuNP ratio of 1.5:1 {0.005 mg/mL BSA and 50.0 nM AuNPs, [NaCl] = 0 mM}) were negative-stained with uranyl acetate and visualized using TEM. TEM imaging clearly shows the binding of BSA to the surface of the AuNPs as white coronas around the individual particles, such as the case with the MEEE-AuNPs and MHA-AuNPs (Figure 3; Figures S9 and S10, Supporting Information). In contrast, negative-stain TEM images of the MPTMA-AuNPs show more loosely associated BSA (elongated white protein fields around the AuNPs) and apparent AuNP-BSA plaques, which are absent in the MEEE-AuNP and MHA-AuNP micrographs (Figure 3D,E, Figures S9 and S10). While caution must be exercised in using an *ex situ* microscopy analysis technique to judge the finer details of the structure of the protein-AuNP conjugates (due to drying and preparation artifacts), the TEM imaging seems to broadly support a picture where BSA is loosely associated with the surfaces of the MEEE- and MHA-AuNPs, while BSA binding to the MPTMA-AuNP surface leads to significant protein misfolding, enough misfolding to drive AuNP-BSA aggregation at this concentration ratio. We therefore characterized the BSA-AuNP conjugates that form in solution more systematically using the absorbance and DLS data.

UV–vis absorbance spectra and DLS data were obtained for BSA-AuNP conjugates formed over a range of [BSA]:[AuNP] concentration ratios (0–50:1 [BSA]:[AuNP] molar ratio). The results of this data are summarized in Figure 3 and Figure S11. In the UV–vis absorbance spectra, the addition of increasing [BSA] has a minimal impact on the shape of the absorbance spectra for the MEEE- and MHA-AuNPs. The primary change in the absorbance spectrum observed is again a small shift in the λ_spr_ once the AuNP surface has been saturated with adsorbed BSA. For the MPTMA-AuNPs, however, the SPR band in the absorbance spectra becomes broader and more shallow, with a raised baseline as the BSA:AuNP molar ratio increases. This is characteristic of the MPTMA-AuNPs forming hetero aggregates with the BSA and other MPTMA-AuNPs as the [BSA] increases.^18,21,26,28^ The DLS data further support this picture as the *D*_h_ for the MPTMA-AuNPs increases to nearly 50 nm, even at a [BSA]:[AuNP] ratio of 0.5:1 (a stoichiometry below surface saturation). The *D*_h_ observed for the MPTMA-AuNPs fluctuates substantially as the [BSA] changes, indicating that the aggregation with BSA may be extensive only at specific BSA:AuNP molar ratios.

The large hydrodynamic diameters (even at low [BSA]:[AuNP] ratios and in a very low ionic strength solution), in conjunction with the TEM images, suggest that binding to the MPTMA-AuNP surface leads to the formation of large BSA-MPTMA-AuNP heteroaggregates. The formation of these extensive aggregates may be correlated to extensive BSA misfolding upon binding. The structure of the BSA-AuNP conjugates for the MPTMA-AuNPs appears to be concentration dependent, with extensive aggregation observed only at specific BSA:AuNP molar ratios. We therefore conclude that (at least at nearly equimolar BSA:AuNP ratios) several (likely 2-3) BSA molecules form a complex with the surface of the MEEE- and MHA-AuNPs under most BSA concentration conditions, while the MPTMA-AuNPs associate with more BSA molecules on a per particle basis and may be prone to heteroaggregate formation with BSA at specific BSA concentrations. These observations are consistent with recent studies that have shown that cationically functionalized nanoparticles (in a variety of size ranges) lead to extensive protein misfolding and heteroaggregation between NPs and protein.^28,30,31^ Thus, the original synthesized surface chemistry of the AuNPs has a profound effect (although a BSA concentration-dependent one) on the biological identity of the BSA-AuNP conjugates.

The characterization of the AuNP-BSA conjugates further leads to the conclusion that absorbance spectroscopy titration and DLS titrations would not be suitable approaches to determine the association constants between the 5 nm AuNPs and BSA. These spectroscopic techniques have previously been used (in conjunction with Langmuir binding isotherm models) to determine affinity constants (*K*_a_) with BSA for large gold nanoparticles (*d*_core_ >20 nm) and gold nanorods.^21,26^ Considering the minimal shift in the SPR wavelength (∼2 nm) for the BSA-AuNP conjugates as [BSA] increases, compounded with the broad, shallow plasmon bands of 5 nm AuNPs, and the aggregation observed for the MPTMA- and MHA-AuNPs, it was clear that absorbance spectroscopy titration did not have the requisite sensitivity to provide a *K*_a_ for the systems under study. This is in contrast to a recent study using 3 and 5 nm citrate-AuNPs, which found that the small citrate-stabilized AuNPs displayed a large shift in the SPR wavelength upon binding to BSA.^20^ Similarly, the concentration-dependent aggregation observed in the binding of BSA to the surfaces of the MPTMA- and MHA-AuNPs would make the affinity constants determined by DLS problematic. To explore the feasibility of absorbance spectroscopy and DLS titration to determine affinity constants on the AuNP surfaces, the absorbance spectroscopy and DLS data for the MEEE-AuNPs (in which no significant aggregation was observed) were fit to a Langmuir isotherm to determine *K*_a_ values for these spectroscopic techniques (Figures S8 and S12, Supporting Information).^42,43^*K*_a_ for BSA adsorbing to MEEE-AuNPs was determined to be 2.4 × 10^5^ and 3.4 × 10^7^ M^–1^ for absorbance and DLS titration experiments, respectively. The same approach did not yield viable *K*_a_ values for the MPTMA- and MHA-AuNPs; aggregation in these systems was too extensive at key [BSA]:[AuNP] ratios. Instead, fluorescence quenching titrations, a technique which has also previously been used to determine *K*_a_ values for BSA binding to large gold nanoparticles, were employed to formally determine the affinity constants for the three AuNP surface chemistries.^21,26,28^

### BSA-AuNP Binding Constant Measurements by Fluorescence Quenching Titration

To determine the affinity constant of BSA for the different AuNP surface chemistries, a fluorescence titration approach (λ_ex_ = 280 nm, λ_em_ = 350 nm) was used to generate a Stern–Volmer plot, which would yield the affinity constant. Then, Hill Plots were constructed to compare the cooperativity of binding between the three AuNP surface chemistries and BSA. Fluorescence quenching titration is generally more useful for determining NP-protein affinity constants than absorbance spectroscopy or DLS titrations, because fluorescence quenching titration does not rely on the monolayer binding assumptions of the Langmuir Isotherm.^21,26,28^ The Langmuir Isotherm traditionally assumes both a small size for the binding species (albumin) relative to the target surface (AuNP) and monolayer coverage of the binding species. These two criteria are unlikely to be met for our system, where the BSA and AuNP have comparable overall sizes, and monolayer coverage by the BSA cannot be taken for granted. Instead, the only key assumption in the fluorescence quenching titration is that the fluorescent BSA and AuNP bind to form a nonfluorescent complex (static quenching).^21,26,28^ To determine if the presence of electrolytes in solution influenced the binding constant, the fluorescence titrations were performed at two ionic strengths, first in Milli-Q deionized water, and then the titration was repeated in 100 mM aqueous sodium chloride. Importantly, because fluorescence titrations are performed with the concentration of the fluorophore (BSA) held constant, and the quencher (AuNP) concentration only has to be increased over a relatively narrow range (1–30 nM in our case) to reach quenching saturation, the fluorescence quenching titrations could be run in concentration ranges where the BSA did not aggregate with the MPTMA- and MHA-AuNPs under study. When AuNP dispersions were combined with a 0.44 mg/mL BSA solution over this concentration range, UV–vis absorbance spectroscopy measurements confirmed no significant SPR broadening and, hence, no aggregation of the AuNPs under the fluorescence quenching titration conditions (Figure S13, Supporting Information).

**Figure 4:**
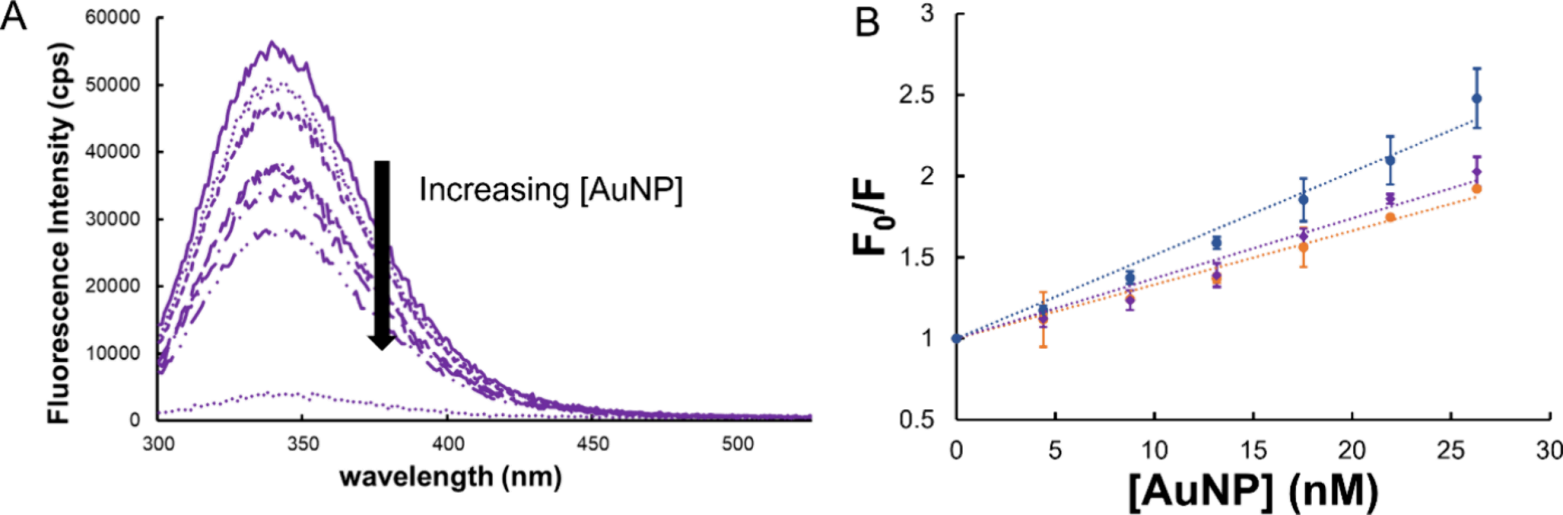
(A) Representative fluorescence emission spectra of bovine serum albumin (BSA) exposed to increasing concentrations of MEEE-AuNPs in 0 mM NaCl. (B) Stern–Volmer plots of fluorescence emission ratios at 350 nm for samples of 0.44 mg/mL BSA incubated at room temperature in Milli-Q deionized water at pH 7.4 with increasing concentrations of: (blue) MHA-capped AuNPs, (purple) MEEE-AuNPs, and (orange) MPTMA-AuNPs. Lines of best fit and standard deviation error bars (*n* = 3) are included.

A representative fluorescence spectrum of aqueous BSA solutions titrated with an increasing concentration of MEEE-AuNPs is shown in Figure 4A. The fluorescence spectra of BSA combined with increasing concentrations of AuNPs show that, as AuNP concentration increases from 0 to 30 nM, fluorescence decreases as a result of quenching by interaction of the excited BSA electrons with the surface of the AuNP. Fluorescence quenching was determined to be saturated at a AuNP concentration of 100 nM. Stern–Volmer plots were then constructed for each AuNP type (Figure 4B), and the slope of the plot was used to determine the affinity constants for BSA and each AuNP surface chemistry. When quenching of the fluorophore is static, that is, when the fluorophore and quencher form a stable complex, *K*_SV_ is equal to the affinity constant (*K*_a_).^21,26,28^ The *K*_a_ determined for each AuNP surface chemistry in the different solution conditions (Milli-Q water and 100 mM NaCl) is shown in Table 2.

**Table 2:** Binding Constants (*K*_a_) for Different AuNP Surface Chemistries Determined by Fluorescence Quenching Titration^a^

	**deionized Water**		**100 mM NaCl**	
**AuNP surface**	*K*_a_ **(× 10**^**7**^**M**^**–1**^**)**	**Hill coefficient (*n*)**	*K*_a_**(× 10**^**7**^**M**^**–1**^**)**	**Hill coefficient (*n*)**
MPTMA	3.3 (±0.16)	1.23 (±0.05)	1.6 (±0.11)	1.34 (±0.13)
MEEE	3.7 (±0.2)	1.30 (±0.07)	3.5 (±0.2)	1.27 (±0.07)
MHA	5.2 (±0.2)	1.15 (±0.09)	4.3 (±0.2)	1.21 (±0.03)

a Deionized water *K*_a_ values determined in Milli-Q water, 25 °C, pH = 7.4. [BSA] = 0.44 mg/mL. Reported errors are based on the standard error in the slope of the line of best fit.

Surprisingly, the *K*_a_ values determined using the fluorescence titration data suggest that electrostatics are not the primary factor in the strength of binding between BSA and the AuNP surface. BSA is largely negatively charged at pH 7.4 (*pI* = 4.7), so it was anticipated that BSA would bind most strongly to the positively charged MPTMA-AuNPs. The extensive aggregation observed when BSA bound to the MPTMA-AuNPs in our DLS and absorbance spectroscopy studies might also support this hypothesis. In fact, the *K*_a_ values were higher for the negatively charged MHA-AuNPs (*K*_a_ = 5.2 × 10^7^ M^–1^), with the MPTMA-AuNPs having a binding constant similar to the neutral MEEE-AuNPs. While the binding constants for the MPTMA, MEEE, and MHA-AuNPs were similar to one another, the binding constants for all of the AuNPs tested were found to be significantly different at the 95% confidence level (two-tailed *t*-test, *p* = 0.05), indicating that the NP surface chemistry does play a significant role in mediating the AuNP-BSA binding interaction, although not exclusively through surface charge.

While electrostatic interactions may not be the primary driving force in the binding of BSA to these small AuNPs, electrostatic interactions do contribute to the strength of the binding interaction, as evidenced by the change in the *K*_a_ value when the fluorescence quenching titration is carried out in water versus 100 mM NaCl. The *K*_a_ values associated with the charged particles (MHA- and MPTMA-AuNPs) decrease when the fluorescence quenching titration is carried out in the salt solution, suggesting that charge screening by the ions is mediating the binding interactions between the AuNP and BSA. However, the *K*_a_ value associated with the MEEE-AuNPs is not significantly different in the salt solution compared to the Milli-Q water (two-tailed *t*-test, *p* = 0.05).

Previously determined values for AuNP-BSA affinity constants vary rather widely depending on the size and shape of the NP.^21,26,28,41,42^ In one instance, in which the binding of BSA to gold nanorods and 20 nm spherical AuNPs was investigated using a similar fluorescence quenching technique, *K*_a_ values ranging from 2.8 to 27.5 nM^–1^ were obtained, depending on the size and shape of the AuNPs under investigation.^21^ For these larger AuNPs, it has generally been found that the affinity constant values increase with NP size, but also that binding strength is strongly correlated with electrostatic contributions (larger positively charged particles should give the highest *K*_a_ values).^21,26,28,41,42^ In other investigations of BSA binding to AuNP surfaces, *K*_a_ values also generally tend to increase with AuNP core size, so the *K*_a_ values obtained in this fluorescence quenching study seem reasonable.^21,26,28^ However, the affinity constant values determined in many studies show a significant variation with the instrumental method employed (particularly in spectroscopic methods versus isothermal titration calorimetry (ITC) measurements on the same system), so a more systematic comparison between the *K*_a_ values obtained here and other studies is challenging.^21,26,28,30−32^ This also makes it challenging to make a meaningful comparison of the *K*_a_ values that we obtained from UV–vis and DLS analysis on the MEEE-AuNPs binding to BSA with the *K*_a_ values that we determined from fluorescence titration. ITC is often the preferred nonspectroscopic technique for determining the affinity constants between nanomaterials and proteins. However, the minimal number of protein-NP interactions that likely occur in 5 nm AuNPs with a protein the size of BSA potentially makes ITC studies impractical for these systems.^21^

A previous study on the effect of citrate-stabilized AuNPs with different sizes has suggested that a model BSA protein corona cannot truly develop on the surface of NPs with core sizes less than 5 nm, due the inability of the smaller particles to induce permanent conformational changes in proteins with comparable sizes.^20^ Our data would also suggest that small AuNPs are unlikely to form strongly bound coronas with proteins the size of albumin and that AuNPs complexed with several (likely 1–3) albumin molecules would be a more accurate picture of the BSA-5 nm AuNP system. We do observe possible evidence of small AuNPs participating in binding interactions that may change the conformation of albumin, though. While the association constant for the MPTMA-AuNPs with BSA is smaller than the *K*_a_ values for the other particle surface chemistries studied, the MPTMA-AuNPs do heteroaggregate readily with BSA at specific BSA concentrations, forming insoluble plaques (which would be consistent with extensive BSA misfolding). Therefore, while the *K*_a_ values obtained here are smaller by approximately an order of magnitude than the *K*_a_ values obtained for BSA binding to large AuNPs using the same techniques, it should be recognized that even the weaker binding interactions may lead to a significant change in protein conformation. It should further be noted that, since the *K*_a_ values we obtained do not point to overall NP surface charge being the primary driving force in BSA adsorption, our data would suggest that specific functional groups present in the amino acid residues of BSA may dominate the binding interaction between BSA and the different AuNP surfaces (at least under these specific conditions). It seems reasonable to assume that van Der Waals forces are the key driving force behind BSA adsorption in this system. This potentially implies that BSA binding to the small AuNPs may change conformation in different ways when binding to each of our AuNP surfaces to maximize contact between the AuNP functional groups and specific amino acid residues within BSA. Previous studies (both experimental and simulated) investigating the binding of albumin (both BSA and HSA) have come to mixed conclusions as to what extent albumin interactions with NP surfaces necessarily lead to conformational changes in the protein.^31,37,42−47^ For instance, a recent computational study found that 4.5 nm AgNPs were unlikely to change HSA conformation during binding events,^46^ although a number of other studies strongly suggest that binding to a NP surface necessarily correlates with changes in protein conformation.^42−45,47^ Further light scattering studies, enzymatic digestion, circular dichroism, and theoretical simulation studies likely will provide much deeper insights into the impact of the small AuNP binding on BSA’s conformation.

**Figure 5:**
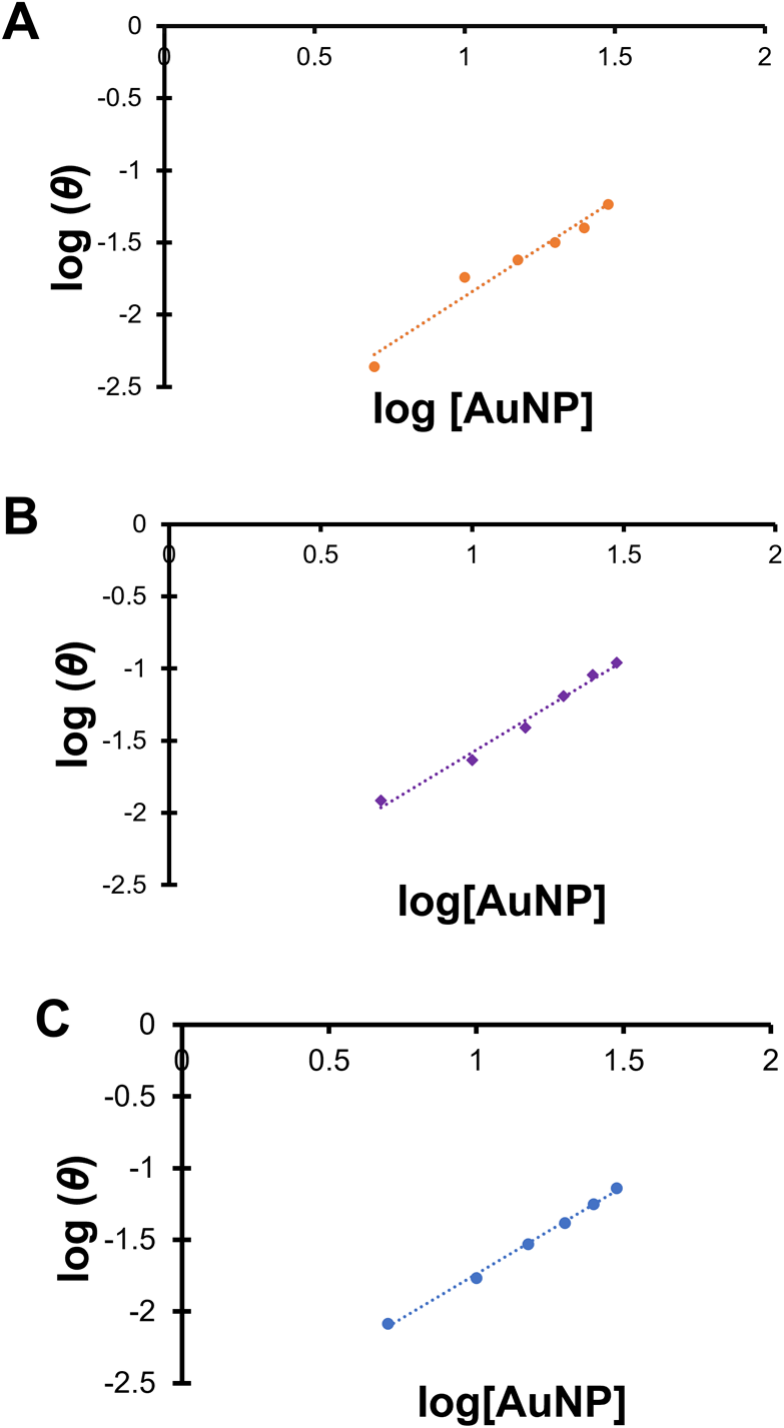
Hill plots of fluorescence emission ratios at λ = 350 nm for samples of 0.44 mg/mL BSA incubated at room temperature in Milli-Q water (*aq*) at pH 7.4, *T* = 25 °C, with increasing concentrations of: (A, orange) MPTMA-AuNPs, (B, purple) MEEE-AuNPs, and (C, blue) MHA-capped AuNPs. Lines of best fit are included. Standard deviation error bars are included but do not extend past the observed data points.

The same fluorescence titration data was also used to determine if the binding of BSA on the NP surface is cooperative or not by using a Hill plot. The log of the ratio (θ) of (*F*_0_*– F*)/(*F – F*_sat_), where *F*_sat_ is the minimum fluorescence emitted due to saturation of the proteins with nanoparticles, was plotted against the log of nanoparticle concentration, and a linear regression was applied.^21^ The slope of this line determines cooperativity: if the slope is <1, the presence of a bound protein molecule decreases the affinity of additional proteins for the nanoparticle, if the slope >1, it increases the affinity, and if the slope is equal to 1, there is no effect in the affinity of additional protein molecules for the nanoparticle. The Hill plots obtained in Milli-Q water are provided below as Figure 5, and the Hill Plots obtained in 100 mM NaCl are provided in the Supporting Information (Figure S15), and the slope of the plots (the Hill coefficients, *n*) are displayed in Table 2. In most cases, the Hill coefficient *n* was greater than 1 (*n* ∼1.3), indicating moderately cooperative binding of BSA molecules to the AuNP surface.

## Conclusions

In this study, we have observed that small (*d*_core_ <10 nm) AuNPs participate in a variety of surface-chemistry-mediated behaviors in their interactions with bovine serum albumin. We used fluorescence quenching titration to determine the affinity constant between BSA and 5 nm AuNPs with different surface chemistries. We find that the electrostatic attraction between the protein and NP does not appear to be the main driving force behind BSA-NP binding events; instead, specific van Der Waals interactions seem to dominate binding strength under this study’s conditions. However, because binding strength is attenuated for positively and negatively charged AuNPs when the fluorescence quenching titration is performed in 100 mM NaCl, there is still some meaningful electrostatic contribution to the binding. Perhaps most interestingly, although the positively charged MPTMA-AuNPs gave the smallest affinity constant values obtained in this study (both in water and in 100 mM NaCl), these particles do appear to interact strongly enough with BSA to induce extensive heteroaggregation (likely correlated with significant protein misfolding) between the AuNPs and BSA at specific BSA:AuNP ratios. Taken together, these results suggest that the original synthetic identity of the small AuNPs can influence the biological identity of the BSA-AuNP conjugate in a variety of ways and reinforce the idea that small AuNPs may possess unique surface chemistry-mediated interactions with proteins of similar size.

## Supporting Information Available

Supplementary absorbance spectroscopy, DLS, and TEM data, as well as fluorescence quenching titration data for the studies run in 100 mM NaCl solutions (PDF)
