# Supplementary material for: Affinity Constants of Bovine Serum Albumin for 5 nm Gold Nanoparticles (AuNPs) with ω-Functionalized Thiol Monolayers Determined by Fluorescence Spectroscopy
Source: Langmuir. 2024 Jul 16;40(30):15558–68. doi: 10.1021/acs.langmuir.4c01234 (PMC11295198; doi:10.1021/acs.langmuir.4c01234)
Supplement: Supplementary file 1 — la4c01234_si_001.pdf [file la4c01234_si_001.pdf]

Supporting Information for "Affinity Constants for Bovine Serum Albumin to 5 nm Gold Nanoparticles (AuNPs) with ω-Functionalized Thiol Monolayers Determined by Fluorescence Spectroscopy"

Jennifer L. Hanigan-Diebel[□], *Robert J. Costin*[†], *Logan C. Myers*[†], *Christopher I. Vandermeer*[†], *Miles S. Willis*[†], *Kiran Takhar*[□], *Ogechukwu V. Odinakachukwu*[□], *Matthias G. Carroll*[□], *Jarrod E. Schiffbauer*[†], *Samuel E. Lohse*[□] [*]

AUTHOR ADDRESSES:

[□]-Chemistry Department, Central Washington University, 400 East University Way, Ellensburg, WA, 98926.

[†]- Department of Physical and Environmental Sciences, Colorado Mesa University, 1100 North Ave, Grand Junction, CO, 81501.

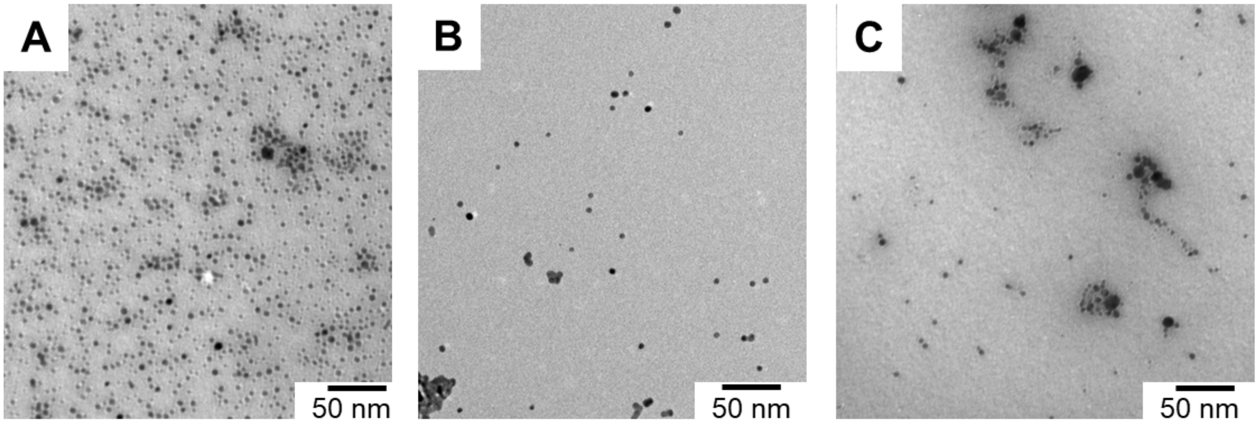

Figure S1. Representative TEM images (300 kV) of: 5 nm (A) MPTMA AuNPs, (B) MEEE AuNPs, and (C) MHA AuNPs. Scale bars are 50 nm.

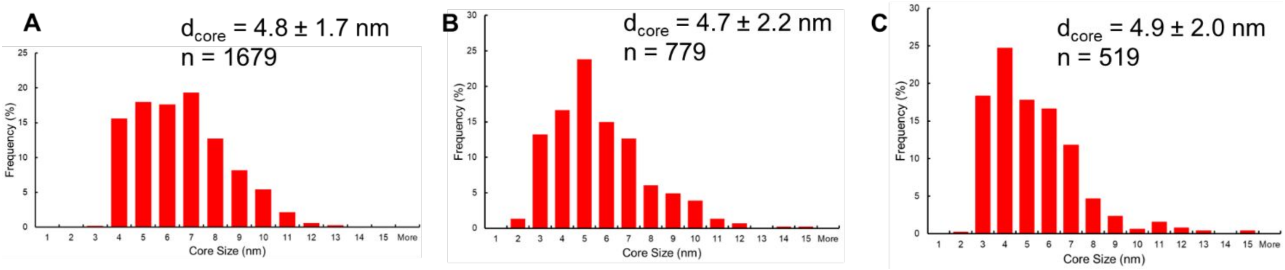

Figure S2. Size distribution histograms (from TEM data) for: (A) MPTMA-AuNPs, (B) MEEE-AuNPs, and (C) MHA-AuNPs.

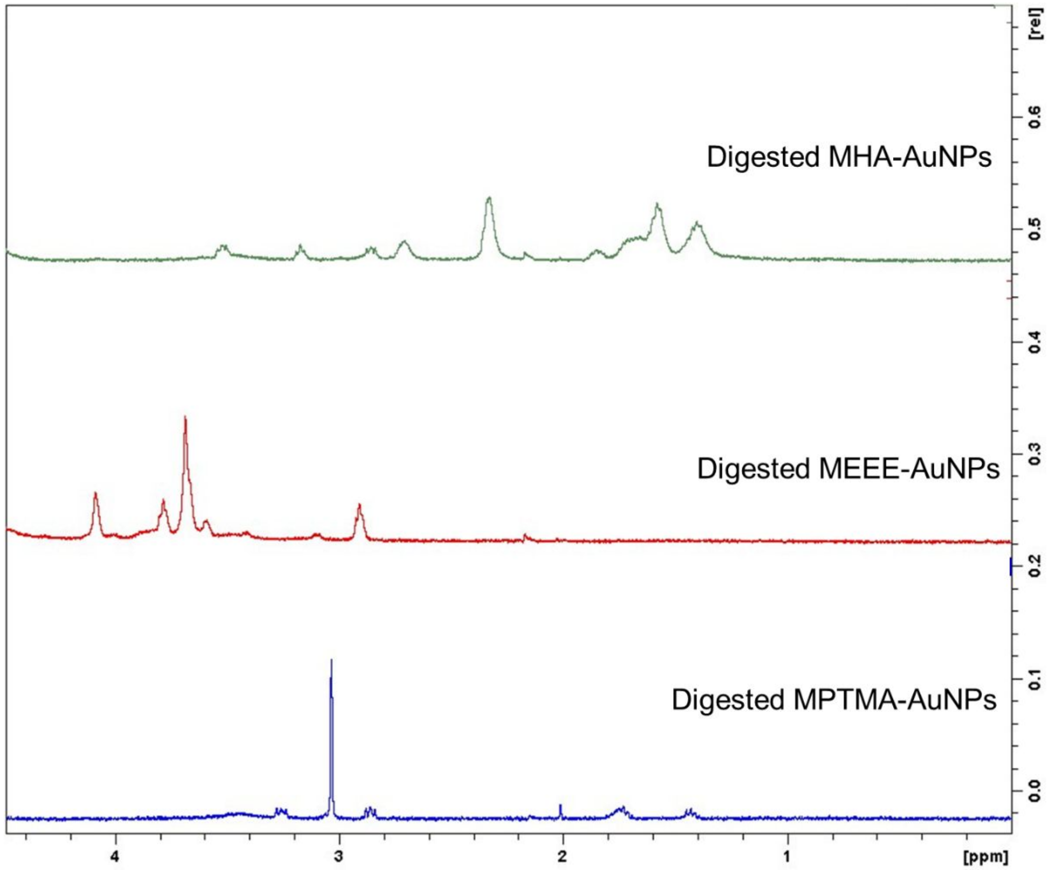

Figure S3. ^{1}H-NMR spectra (400 MHz) of diiodide-digested MHA-capped, MEEE-capped, and MPTMA-capped AuNPs in deuterium oxide (D_2O).

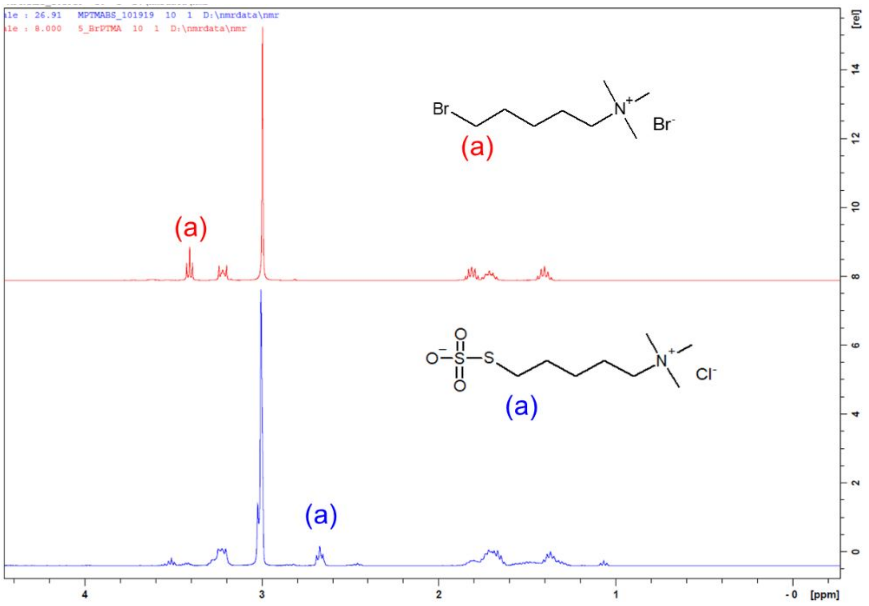

Figure S4. ^{1}H-NMR spectra (400 MHz) of (5-bromopentyl)trimethylammonium bromide and the Bunte salt analog of mercapto pentyltrimethylammonium chloride (D_2O).

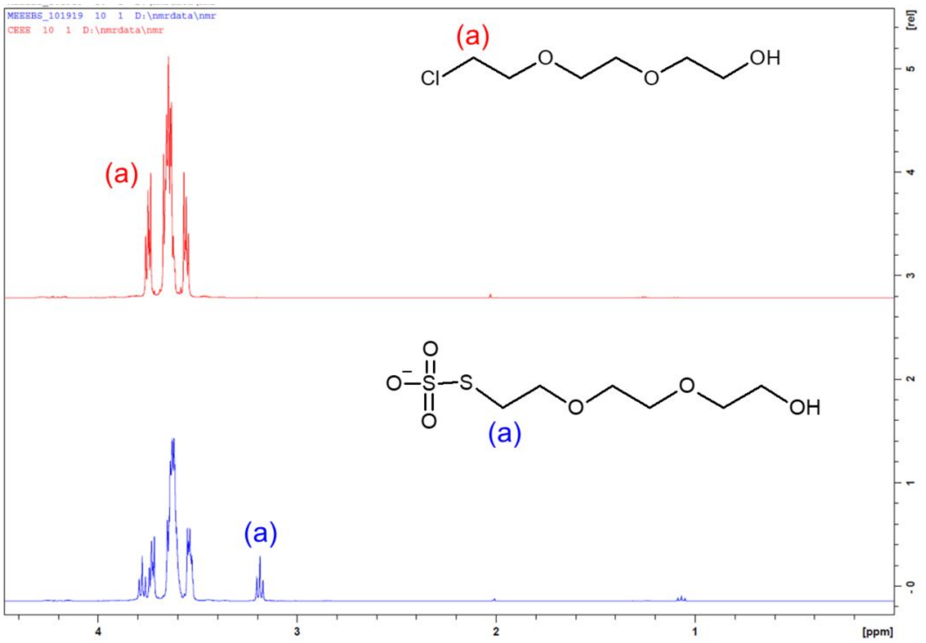

Figure S5. ^{1}H-NMR spectra (400 MHz) of 2-[2-(2-chloroethoxy)ethoxy]ethanol and the Bunte salt analog of mercapto ethoxyethoxyethanol (D_2O).

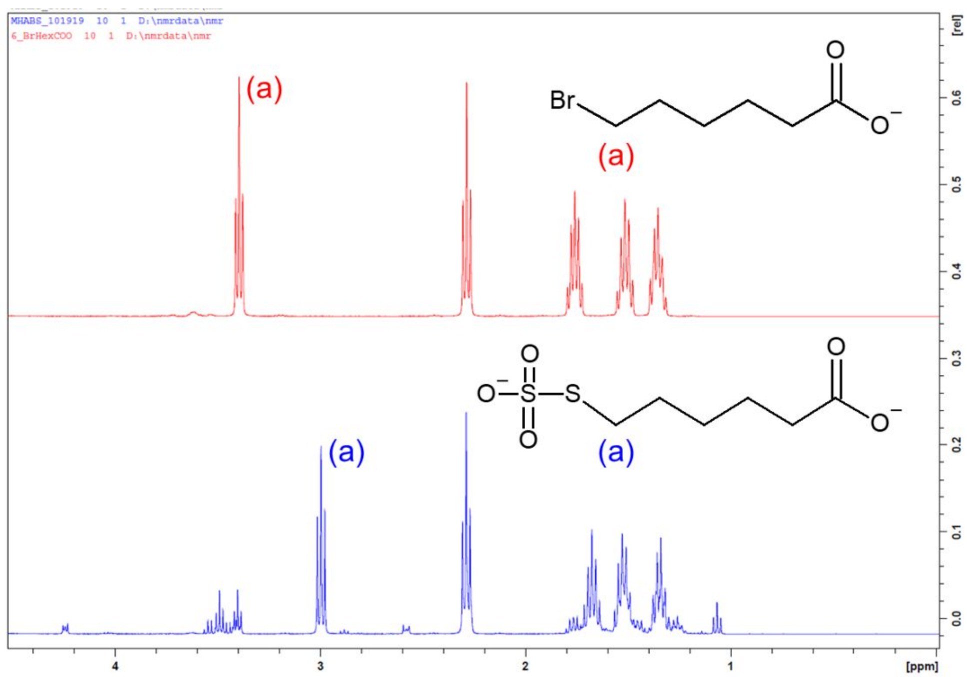

Figure S6. ^{1}H-NMR spectra (400 MHz) of 6-bromohexanoic acid and the Bunte salt analog of mercapto hexanoic acid (D_2O).

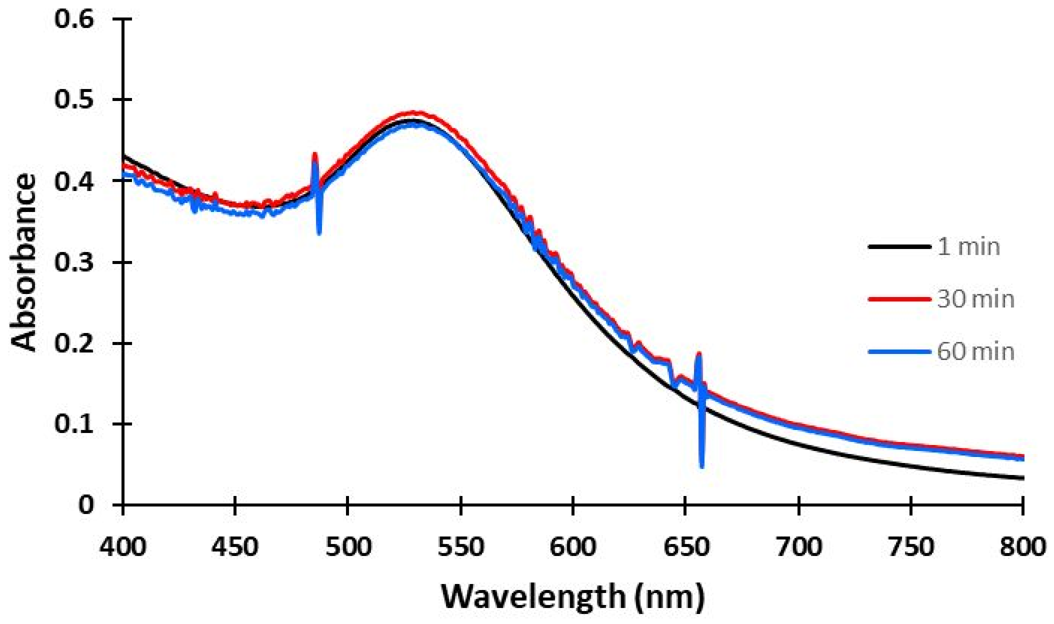

Figure S7. Time-dependent UV-vis absorbance spectra of MHA-capped AuNPs incubated in 30 mg/mL BSA and 100 mM NaCl at pH 7.4 from 0 to 60 minutes of incubation at 25 ˚C.

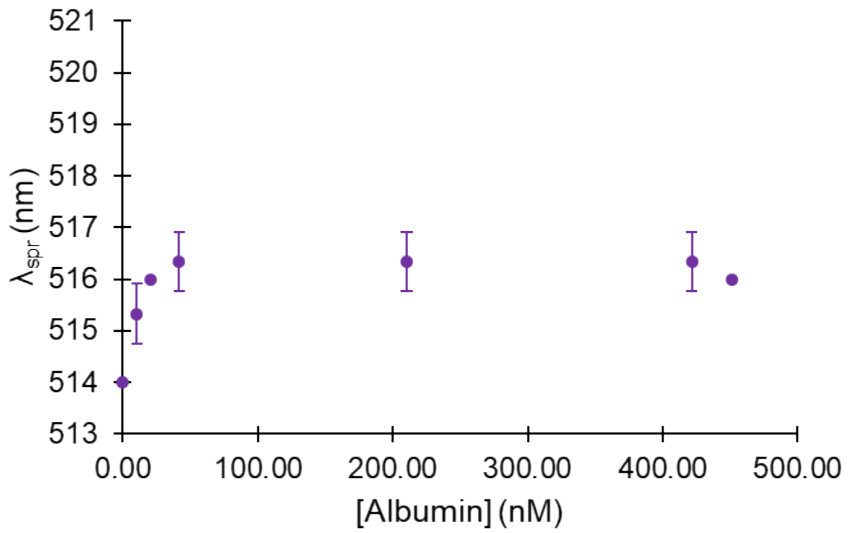

Figure S8. Absorbance titration data for MEEE-AuNPs titrated with increasing concentrations of BSA at 25 ˚C, pH = 7.4, [NaCl] = 0 mM, [AuNP] = 50 nM. Standard deviations are based on three trials ($n = 3$).

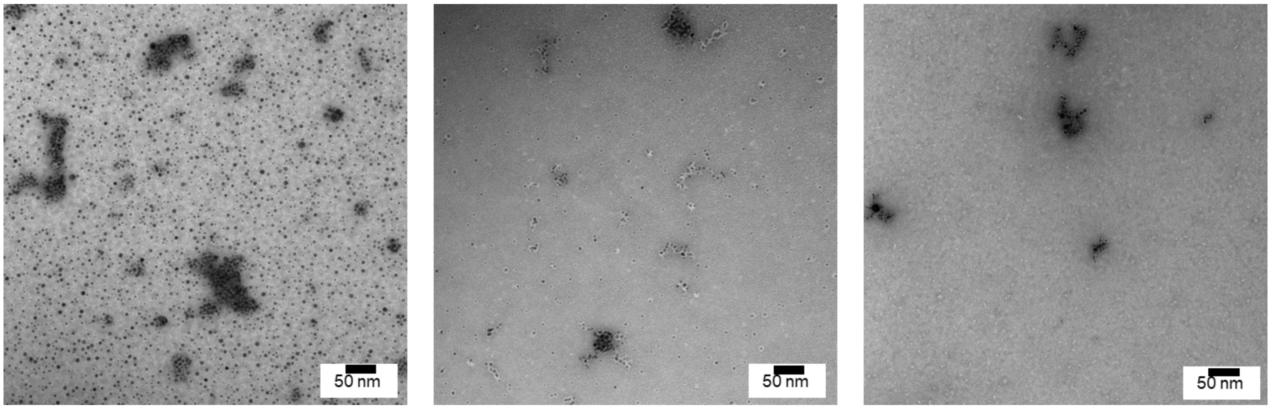

Figure S9. Representative uranyl-acetate stained TEM images (300 kV) of BSA-AuNP complexes of (A) MPTMA AuNPs, (B) MEEE AuNPs, and (C) MHA AuNPs. Scale bars are 50 nm.

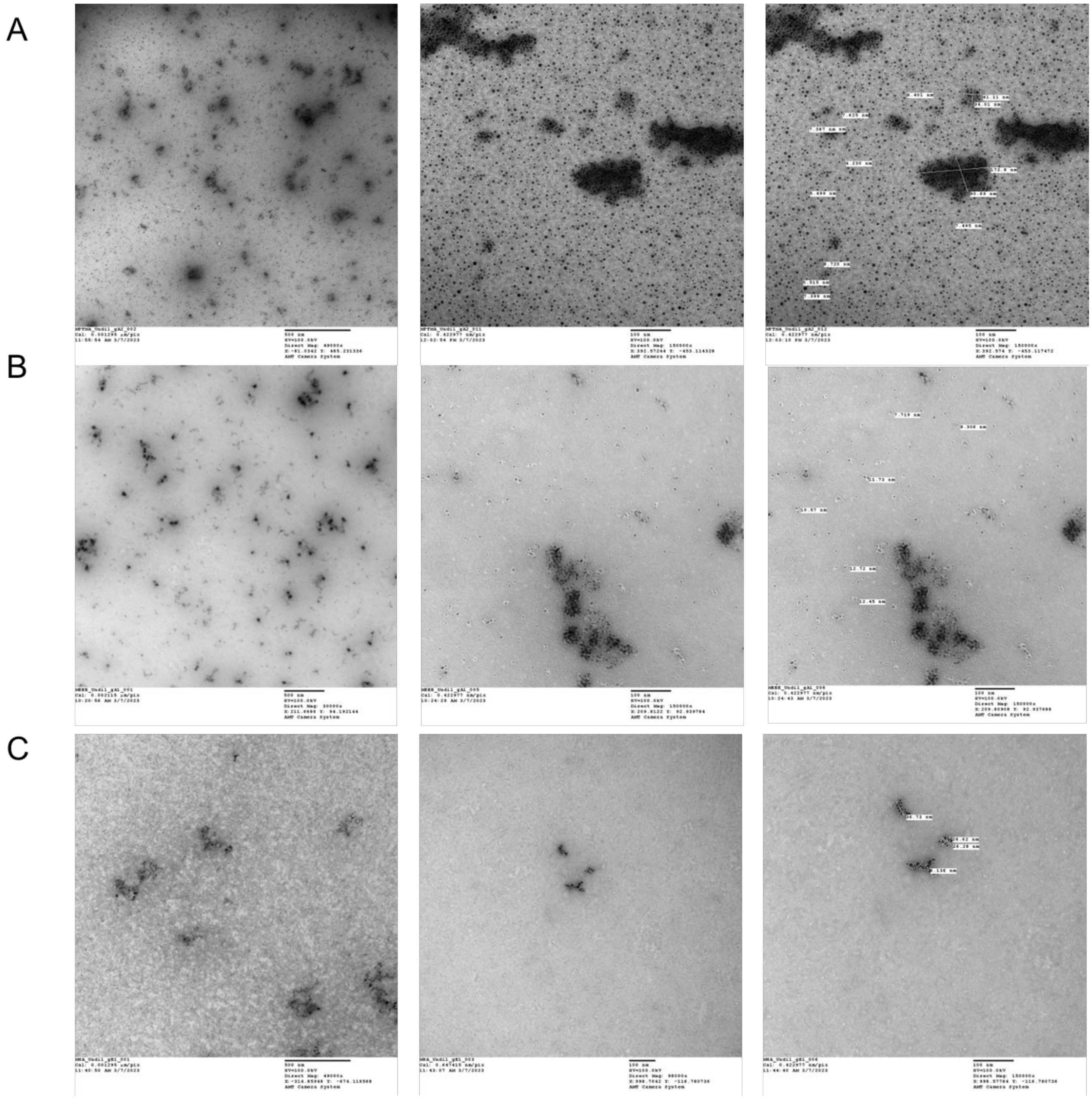

Figure S10. Low and enhanced magnification (with cluster measurement) uranyl-acetate stained TEM images (300 kV) of BSA-AuNP complexes of (A) MPTMA AuNPs, (B) MEEE AuNPs, and (C) MHA AuNPs. Scale bars are 500 nm in the low magnification images (Left column) and 100 nm in the high magnification images (Center and Right columns).

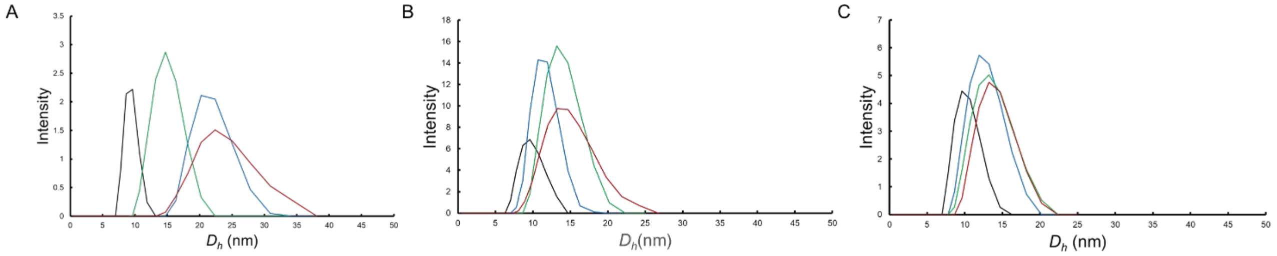

Figure S11. Dynamic light scattering intensity data related to (A) MPTMA AuNPs, (B) MEEE AuNPs, and (C) MHA AuNP dispersion upon exposure to different [BSA] at pH = 7.4. Black trace AuNP dispersion prior to BSA exposure, blue trace (0.5:1 BSA:AuNP molar ratio), green trace (1.5:1 BSA:AuNP), and red trace (50:1 BSA:AuNP).

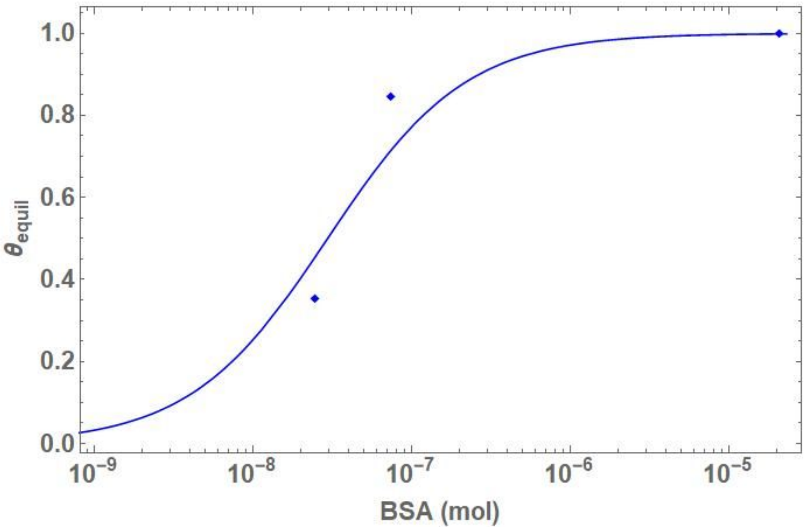

Figure S12. DLS titration data for MEEE-AuNPs titrated with increasing concentrations of BSA at 25 ˚C, pH = 7.4, [NaCl] = 0 mM, [AuNP] = 50 nM.

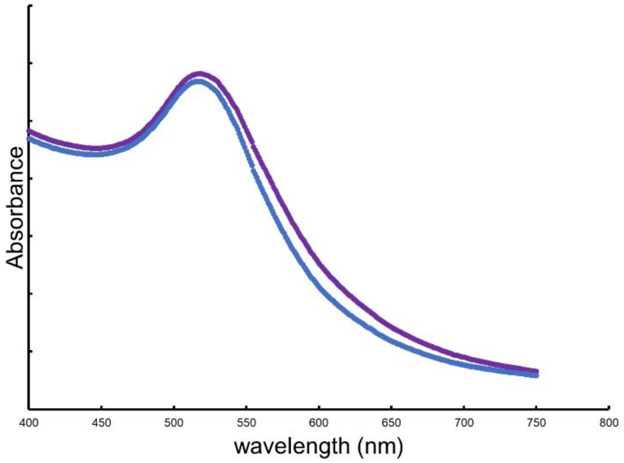

Figure S13. Time-dependent UV-visible absorbance spectra of MPTMA AuNPs (orange), MEEE AuNPs (purple) and MHA AuNPs (blue), each in 0.44 mg/mL BSA, [AuNP] = 30 nM, at pH 7.4 after 180 minutes of incubation time. Note that the MPTMA and MHA AuNP absorbance traces almost perfectly overlay.

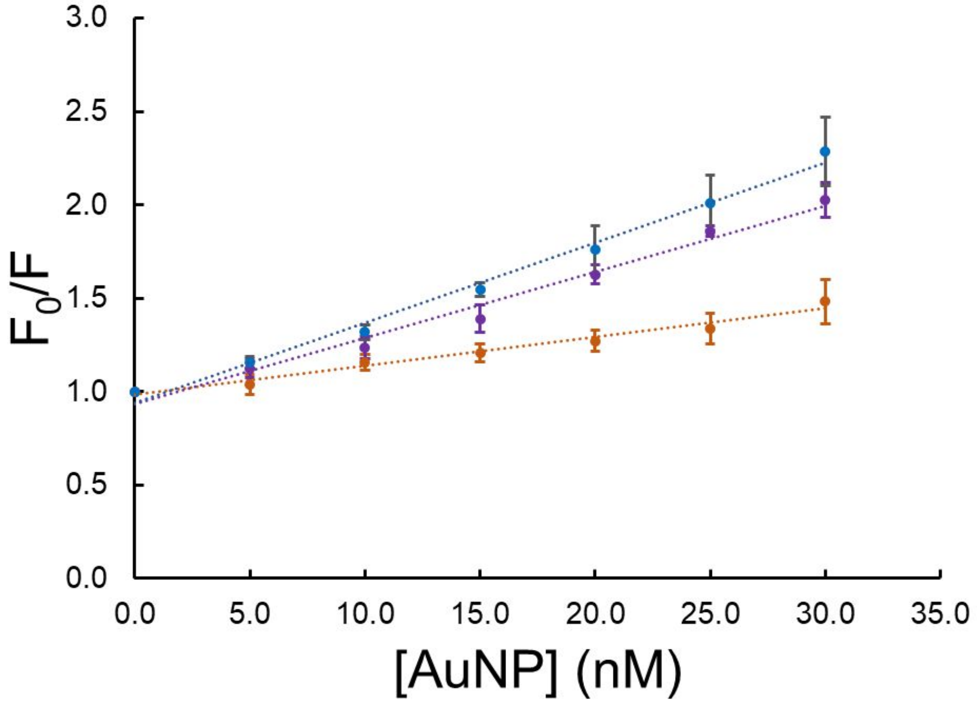

Figure S14. Stern-Volmer plots of fluorescence emission ratios at 350 nm for samples of 0.44 mg/mL BSA incubated at room temperature in 100 mM NaCl(*aq*) at pH 7.4 with increasing concentrations of: (Blue) MHA-capped AuNPs, (Purple) MEEE AuNPs, and (Orange) MPTMA AuNPs. Lines of best fit and standard deviation error bars ($n = 3$) are included.

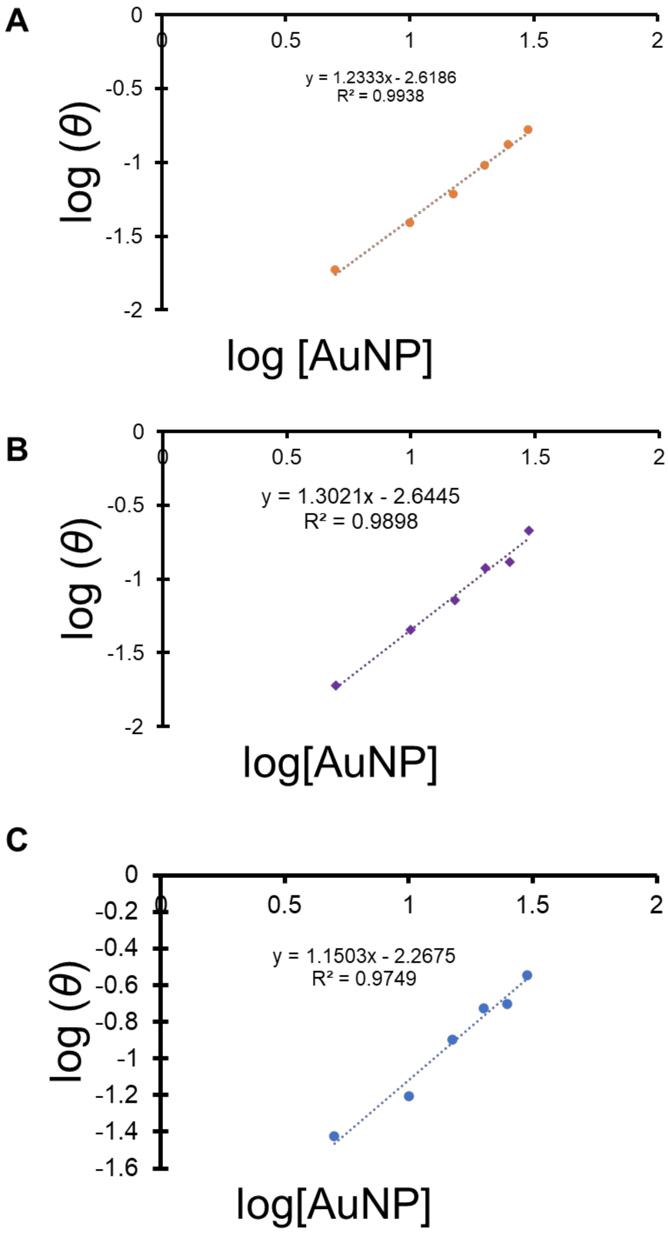

Figure S15. Hill plots of fluorescence emission ratios at 350 nm for samples of 0.44 mg/mL BSA incubated at room temperature in 100 mM NaCl(*aq*) at pH 7.4, T = 25 ^{0}C, with increasing concentrations of: (A) MPTMA AuNPs, (B) MEEE AuNPs and (C) MHA-capped AuNPs. Lines of best fit are included.

Langmuir Adsorption Isotherm for Absorbance Spectroscopy and DLS Titration.

To determine the value of the BSA-MEEE-AuNP affinity constant (K_a), the BSA concentration and the corresponding hydrodynamic diameter (D_h) was fit to a Langmuir binding isotherm, using the following equation:[1]

$$\frac{\Delta D_h}{\Delta D_{max}} = \frac{K_a[\text{BSA}]}{1+K_a[\text{BSA}]} \qquad \text{(S1)}$$

Here, ΔD_h is the change in the mean hydrodynamic diameter, and ΔD_h(max) is the overall change in the mean hydrodynamic diameter between the initial conditions and saturation. The same basic Langmuir isotherm was used to determine the K_a value based on the change in absorbance wavelength ($\Delta\lambda_{SPR}$) from the UV-vis absorbance titration data. In this model, $\Delta\lambda$(max) is again the change in the SPR wavelength between the initial conditions and saturation.[1]

$$\frac{\Delta\lambda_{SPR}}{\Delta\lambda_{max}} = \frac{K_a[\text{BSA}]}{1+K_a[\text{BSA}]} \qquad \text{(S2)}$$

REFERENCES

1. Yang, J.A.; Johnson, B.J.; Wu, S.; Woods, W.S.; George, J.M.; Murphy, C.J. Study of Wild-Type α-Synuclein Binding and Orientation on Gold Nanoparticles. *Langmuir* **2013**, *29*, 4603-4615. DOI:10.1021/la400266u
